# Multicenter cohort study on duration of antiarrhythmic medication for supraventricular tachycardia in infants

**DOI:** 10.1007/s00431-022-04757-5

**Published:** 2022-12-28

**Authors:** Minna Mecklin, Anniina Linnanmäki, Anita Hiippala, Topias Leino, Anita Arola, Markku Leskinen, Hanna Ruotsalainen, Juha-Matti Happonen, Tuija Poutanen

**Affiliations:** 1 Paediatric Cardiology, Department of Paediatrics, Tampere University Hospital, Tampere, Finland; 2 Tampere Center for Child, Adolescent and Maternal Health Research, Faculty of Medicine and Health Technology, Tampere University, Tampere, Finland; 3 Paediatric Cardiology, Department of Paediatrics and Adolescent Medicine, Helsinki University Hospital, Helsinki, Finland; 4 Paediatric Cardiology, Department of Paediatrics and Adolescent Medicine, Turku University Hospital, Turku, Finland; 5 Paediatric Cardiology, Department of Paediatrics and Adolescence, Oulu University Hospital, Oulu, Finland; 6 Paediatric Cardiology, Department of Paediatrics, Kuopio University Hospital, Kuopio, Finland

**Keywords:** Supraventricular tachycardia, Atrioventricular reentrant tachycardia, Infants, Antiarrhythmic medication, Prophylaxis

## Abstract

Antiarrhythmic medication (AM) is commonly used to prevent supraventricular tachycardia (SVT) recurrence in infants. Our aim was to determine whether a shorter duration of AM is sufficient to prevent atrioventricular reentrant tachycardia (AVRT) recurrence and evaluate risk factors for recurrence of SVT after discontinued AM.This multicenter cohort study included all infants diagnosed with SVT in the five university hospitals in Finland between 2005 and 2017. Those diagnosed between 2005 and 2012 received AM for 12 months (group 1), and those diagnosed between 2013 and 2017 received AM for 6 months (group 2). A total of 278 infants presented with AVRT (group 1, *n* = 181; group 2, *n* = 97), and the median AM duration was 12.0 months (interquartile range [IQR] 11.4–13.4) and 7.0 months (IQR 6.0–10.2), respectively. Propranolol was the most frequently used first-line AM (92% and 95%). Recurrence-free survival rates were over 88% until 12 months after AM prophylaxis in both groups, without any statistically significant difference between them. Independent risk factors for recurrence of SVT after discontinuation of AM were need of combination AM (*HR* 2.2, *95% CI* 1.14–4.20), Wolff-Parkinson-White (WPW) syndrome (*HR* 2.4, *95% CI* 1.25–4.59), and age over 1 month at admission (*HR* 2.2, *95% CI* 1.12–4.48).

*    Conclusion*: Shortening AM duration in infants from 12 to 6 months does not seem to lead to more frequent SVT recurrence. The risk factors for recurrence of SVT were WPW syndrome, need of combination AM, and age over 1 month.

**Supplementary Information:**

The online version contains supplementary material available at 10.1007/s00431-022-04757-5.

**Table:** 

**What is Known:** *• Despite the evidence of spontaneous resolution of supraventricular tachycardia episodes in infants, the antiarrhythmic medication is often continued for up to twelve months.* *• The optimal approach for duration of antiarrhythmic medication remains unknown.*.	
**What is New:** *• Shortening the duration of antiarrhythmic medication in infants with supraventricular tachycardia does not seem to increase the risk of subsequent recurrence.* *• Infants whose arrhythmia was successfully controlled with monotherapy were the group least likely to have recurrence after discontinuation of antiarrhythmic medication.*	

## Introduction

Supraventricular tachycardia (SVT) is the most common cardiac arrhythmia in children [1]. In infants, SVT is most frequently caused by an accessory pathway, leading to atrioventricular reentrant tachycardia (AVRT) [1, 2]. Approximately 60% of pediatric patients with SVT experience their first episode during first year of life and 38% during first two months [3, 4]. To prevent heart failure and mortality, infants with SVT are treated prophylactically with antiarrhythmic medication (AM) as long as the risk of recurrent SVT exists [2]. Fortunately, the risk decreases spontaneously with age; more than 90% of infants with SVT will cease having episodes of tachycardia by 8 months of age [4]. However, the time frame for spontaneous resolution of SVT and the optimal approach for AM in infants remains unknown, and in practice, the duration of AM needs to be tailored on a case-by-case basis and varies considerably up to 12 months [2, 5–7]. Only a few studies have reported the duration of SVT prophylaxis [2, 7] or evaluated the impact of duration on the risk of SVT recurrence [8]. A double-blind multicenter randomized controlled trial on AM in infants with SVT (SAMIS) compared digoxin and propranolol. It was underpowered to reveal differences in efficacy between the two AMs [6]. The most important finding in that study was that during the 12 months’ follow-up, no new recurrence of SVT occurred after 110 days. Until 2013, the Finnish national practice for SVT secondary prophylaxis in infants was to use AM for 12 months. As the SAMIS study suggested that the duration of AM might be unnecessarily long, we shortened it to 6 months in January 2013.

The aim of this study was to evaluate and compare the recurrence of SVT in infants treated for 6 months with AM in 2013–2017 and infants treated for 12 months with AM in 2005–2012.

## Materials and methods

We retrospectively reviewed the patient charts of infants diagnosed with SVT between 2005 and 2017. We collected the data from the patient registry databases of the five university hospitals in Finland (Helsinki, Tampere, Turku, Kuopio, and Oulu). These hospitals provide secondary care for those living in their primary catchment areas, which together cover over 60% of the Finnish infant population under 12 months of age, and tertiary care for the entire country.

The collected data included medical history, antenatal arrhythmias (and AMs if present), physical examination and echocardiogram findings, acute treatments of arrhythmias (including detailed information on AM used), electrocardiography (ECG) and Holter monitoring findings at diagnosis and during follow-up, respiratory support and intensive care if needed, and recurrence of arrhythmia after AM. The information on characteristics and medication was categorized in bivariable (yes and no). If the information was not mentioned in medical records, the variable was categorized in category no. Infants born before 37 gestational weeks were categorized premature. Left ventricular dysfunction was defined as ejection fraction less than 50% or fractional shortening less than 28%. CHD was sorted hierarchically, as previously presented [9].

The diagnoses of SVT and arrhythmia recurrence were based on ECG or Holter monitoring findings. We included infants diagnosed with AVRT in the study, and other types of supraventricular arrhythmias (atrial flutter, ectopic atrial tachycardia, persistent junctional reciprocating tachycardia) were excluded. The AVRT diagnosis was based on ECG. Wolff-Parkinson-White (WPW) syndrome was diagnosed if ventricular preexcitation was detected at least once in ECG. We considered SVT in patients without preexcitation as AVRT acknowledging the fact that a small fraction of the patients might actually have had atrioventricular nodal reentrant tachycardia (AVNRT). Patients who died of a reason other than arrhythmia or who dropped out after acute treatment without any follow-up data available were excluded.

In the present study, recurrence of SVT was defined as the first SVT episode after finishing AM prophylaxis. Breakthrough episodes during AM were addressed as inadequate therapy. Outpatient visits during follow-up included ECG and Holter monitoring at 1, 3, and 6 months and 1 month after termination of AM. Those who continued AM for over 6 months were invited to a follow-up visit at 12 months.

The national practice for SVT treatment in Finland was changed in 2013. Until 2012, infants with SVT were treated with prophylactic AM for 12 months. When AM was continued for 12 months, the dose was adjusted according to the current body weight or surface area during the first six months, and after that, weight-based dosing corrections were no longer performed. At the beginning of 2013, the practice was changed, and AM prophylaxis was reduced to 6 months. In the present study, the patients were divided into two groups according to year of SVT diagnosis: patients diagnosed with SVT between 2005 and 2012 (group 1, planned AM prophylaxis for 12 months) and patients diagnosed with SVT between 2013 and 2017 (group 2, planned AM prophylaxis for 6 months).

We compared the incidence of SVT recurrence after AM prophylaxis in the two groups, which had been treated with AM prophylaxis for either a long (group 1) or short (group 2) duration. The primary outcomes of the study were the incidence of SVT recurrence after discontinuation of AM prophylaxis after 6 or 12 months and the recurrence-free survival rate over time. In addition, we evaluated the risk factors for recurrence of SVT after discontinuation of AM.

### Statistics

IBM SPSS Statistics 26 (Armonk, NY) was used for all statistical analyses. The Mann–Whitney *U* test or Kruskal–Wallis test was used for non-normally distributed continuous variables, and the results presented as medians and interquartile ranges (IQRs). We used Shapiro–Wilk methods to test of normality of a distribution. A *t*-test was used for the normally distributed continuous variables, and the results presented as means and 95% confidence intervals (95% Cl). *χ*^2^ or Fisher’s exact tests, when appropriate, were used for categorized variables, and the results expressed as numbers and percentages. A two-sided *p* value below 0.05 was considered statistically significant. We used the Kaplan–Meier method to express recurrence-free survival and the log-rank test for comparing the two groups. The Cox regression model was used in multivariate analysis, and the results expressed as hazard ratios (HRs) and 95% CI. Variables with *p* values less than 0.2 in the univariate analysis were included in the multivariate analysis.

### Ethics

The study was approved by the medical director of each of the five university hospitals. According to Finnish legislation, registry-based studies do not need ethics committee approval.

## Results

From 2005 to 2017, 397 infants younger than 12 months were diagnosed with supraventricular arrhythmias. Children who died due to reasons other than arrhythmia (*n* = 4), dropped out after acute treatment (*n* = 43), or had arrhythmia caused by mechanisms other than AVRT (*n* = 72) were excluded; thereafter, 278 infants who had AVRT were included in the analyses. Their median age was 8 days (*IQR* 1–18, Table 1) at presentation. CHD was present in 16% of the patients (*n* = 44), and the types of CHD are listed in Table S1. Antenatal arrhythmia was diagnosed in 24% of the patients and half of them had been treated with antenatal AM. WPW syndrome was more common in group 1 (24% vs. 9%, *p* = 0.002), and the majority (87%) showed ventricular preexcitation in their first ECG on sinus rhythm (Table 1). Among the 52 infants with ventricular preexcitation, it was still present in 23 (44%) at the age of 1 year.

**Table 1:** Demographic data and clinical findings at the time of diagnosis of 278 infants diagnosed with SVT presented in two groups, those diagnosed between 2005 and 2012 and those between 2013 and 2017

	Total *n* = 278	2005–2012 *n* = 181	2013–2017 *n* = 97	*p* value
Age (days, median, *IQR*)	8 (1–18)	7 (1–17)	9 (1.5–24)	0.119
Age, *n* (%)				0.365
< 1 month	230 (83)	154 (85)	76 (78)	
1–3 month	33 (12)	17 (9.4)	16 (17)	
4–6 months	8 (2.9)	5 (2.8)	3 (3.1)	
7–12 months	7 (2.4)	5 (2.8)	2 (2.1)	
Sex (male, %)	172 (62)	114 (63)	58 (60)	0.607
CHD, *n* (%)	44 (16)	27 (15)	17 (18)	0.332
Prematurity, *n* (%)	53 (19)	34 (19)	19 (20)	0.348
Antenatal arrhythmia, *n* (%)	66 (24)	43 (24)	23 (24)	0.993
Antenatal AM, *n* (%)	31 (11)	23 (13)	8 (8.2)	0.276
WPW syndrome, *n* (%)	52 (19)	43 (24)	9 (9.3)	0.002
Delta wave in primary sinus ECG	45 (16)	38 (21)	7 (7.2)	0.002
Intermittent delta wave	29 (10)	24 (13)	5 (5.2)	0.022
Persistent delta wave	23 (8.3)	19 (11)	4 (4.1)	0.041
Congestive heart failure, *n* (%)	74 (27)	51 (28)	23 (24)	0.478
Inotropic medication, *n* (%)	51 (18)	35 (19)	16 (17)	0.702
Respiratory support, *n* (%)	63 (23)	43 (24)	20 (21)	0.502
NIV	19 (6.8)	9 (5.0)	10 (10)	
Invasive ventilation	44 (16)	34 (19)	10 (10)	

SVT supraventricular tachycardia, IQT interquartile range, CHD congenital heart disease, AM antiarrhythmic medication, WPW Wolff-Parkinson-White syndrome, NIV non-invasive ventilation

Except for ventricular preexcitation, there were no other statistically significant differences in the basic demographics or disease severity (Table 1) of the infants diagnosed with SVT between 2005 and 2012 (group 1) and those diagnosed between 2013 and 2017 (group 2). At admission, 27% of the infants had congestive heart failure, and 17% had left ventricular dysfunction. Eight hemodynamically unstable children needed resuscitation upon admission. One patient had cardiovascular collapse during intravenous amiodarone infusion, leading to resuscitation and death. One patient in group 1 required a left ventricular assist device, and one patient in group 2 required extracorporeal membrane oxygenation treatment.

Acute treatment at admission and selected prophylactic AM data are presented in Table 2. Adenosine was used in more than half of the patients in both groups. Over 80% responded to adenosine alone (group 1, *n* = 85 [83%]; group 2, *n* = 44 [90%]). Nearly all (98%) of the infants received AM as a secondary prophylaxis, i.e., after the SVT diagnosis. In both groups, over 90% of infants received propranolol, and it was the first-line medication in 83% of cases (Table 2). The median dose of propranolol was 3.4 mg/kg/day (*IQR* 3.0–4.0). At the age of 6 months, median dose of propranolol was 3 mg/kg/day (*IQR* 2.6–3.4) and 2.8 mg/kg/day (*IQR* 2.5–3.0) in groups 1 and 2, respectively. The propranolol dose was not adjusted for weight after age of 6 months in group 1 and the median dose of propranolol at age of 12 months was 2.6 mg/kg/day (*IQR* 2.2–3).

**Table 2:** Acute treatment and prophylactic antiarrhythmic medication in infants diagnosed with SVT presented in two groups, those diagnosed between 2005 and 2012 and those between 2013 and 2017

		2005–2012, *n* = 181		2013–2017, *n* = 97	
		Acute treatment	Prophylaxis	Acute treatment	Prophylaxis
Synchronized cardioversion, *n* (%)		10 (5.5)		1 (1.0)	
Adenosine, *n* (%)		102 (56)		49 (51)	
Propranolol, *n* (%)		10 (5.5)	166 (92)	4 (4.1)	92 (95)
Flecainide or propafenone, *n* (%)		2 (1.1)	36 (20)^a^	1 (1.0)	17 (18)^b^
Amiodarone, *n* (%)		17 (9.4)	26 (14)	4 (4.1)	20 (21)
Sotalol, *n* (%)			39 (22)		12 (12)
Other^c^, *n* (%)			10 (5.5)		2 (2.1)
No medication, *n* (%)			5 (2.8)		0

SVT, supraventricular tachycardia

^a^Flecainide n=32, propafenone n=4

^b^Flecainide n=14, propafenone n=3

^c^Other medication such as atenolol, metoprolol, or digoxin

Over 60% of the infants (*n* = 169) were successfully treated with monotherapy, mostly propranolol (Table 3). One-third needed a combination of two AMs in both groups. The median duration of AM was 12.0 months (*IQR* 11.4–13.4) in group 1 and 7.0 months (*IQR* 6.0–10.2, *p* < 0.0001) in group 2. Eight infants (3%) had repeated breakthrough arrhythmias, and they continued to receive AM throughout the entire follow-up period. The duration of their AM was between 4.5 and 14.4 years.

**Table 3:** Prophylactic antiarrhythmic medication (AM) in supraventricular tachycardia in infants presented in two groups, those diagnosed between 2005 and 2012 and those between 2013 and 2017

	2005–2012 *n* = 176	2013–2017 *n* = 97	*p* value
Successful monotherapy, n (%)	106 (59)	63 (64)	0.515
Propranolol	97 (54)	58 (60)	
Sotalol	7 (3.9)	2 (2.1)	
Amiodarone	2 (1.1)	3 (3.1)	
More than one AM used alone, *n* (%)	16 (8.8)	4 (4.1)	0.152
Combination of two AMs, *n* (%)	47 (31)	26 (31)	1.000
Propranolol and flecainide	23 (13)	12 (12)	
Propranolol and amiodarone	14 (7.7)	11 (11)	
Propranolol and other	6 (3.3)	1 (1.0)	
Sotalol and flecainide/amiodarone	4 (2.2)	2 (2.0)	
Combination of three or more AMs, *n* (%)	5 (2.8)	2 (2.1)	1.000
More than one different combination, *n* (%)	2 (1.1)	2 (2)	0.614

Infants with WPW syndrome (*n* = 52) were treated similarly to infants with concealed pathway. Propranolol was initial AM in 46 infants (88%) with no differences in groups 1 and 2 (*p* = 0.965), and monotherapy was successful in 25 infants (48%). The median dose of propranolol was 3.5 mg/kg/day (*IQR* 3.2–4) in infants with ventricular preexcitation. There was no difference in median dose between infants with or without WPW syndrome (*p* 0.082). Furthermore, the median duration of AM was 12.0 months (*IQR* 10.7–13.4) and 11.6 (*IQR* 7.6–12.7) in infants with delta wave and those with concealed pathway, respectively (*p* = 0.081).

After AM was discontinued, 44 (16%) infants with SVT experienced recurrences during the follow-up: 34 (19%) in group 1 and 10 (10%) in group 2 (*p* = 0.053). In both groups, over 70% of the recurrences occurred during the first year after discontinuing AM. The recurrence rates during the first year after AM discontinuation were 11% (*n* = 20) and 10% (*n* = 10) in groups 1 and 2, respectively. None of the children had congestive heart failure at the time of recurrence. The median time for recurrence after discontinuing AM was 7.1 months (*IQR* 1.2–46) in group 1 and 1.8 months (*IQR* 0.6–5.4, *p* = 0.147) in group 2. The median follow-up time was 10.3 years (*IQR* 8.7–12.2) in group 1 and 4.5 (*IQR* 3.0–5.6) in group 2.

**Fig. 1:**
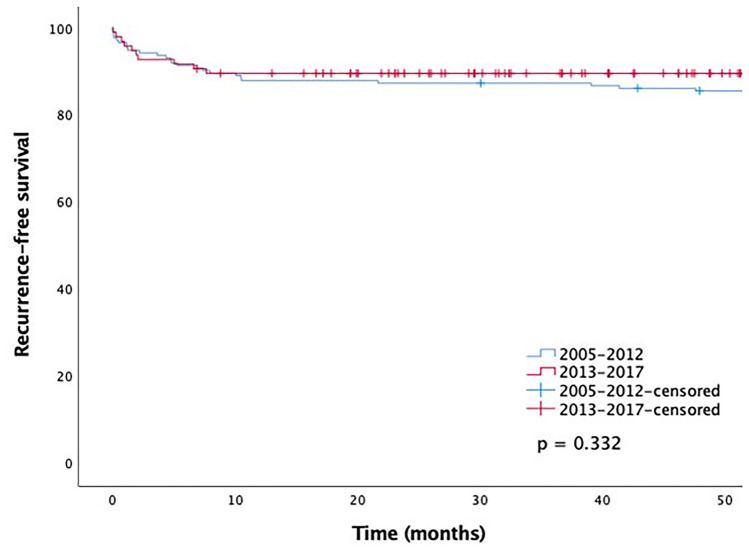
Recurrence-free survival after discontinuation of antiarrhythmic medication in the two groups of infants with supraventricular tachycardia in 2005–2012 and 2013–2017

The recurrence-free survival rate was 92% in both groups 6 months after discontinuing AM and 88% and 90% 12 months after discontinuing AM in groups 1 and 2, respectively. There was no statistically significant difference between the recurrence-free survival rates of the two groups (Fig. 1). We included clinically and statistically significant variables in the Cox regression multivariate analysis. When age, sex, and presence of ventricular preexcitation were included in the model, the shorter duration of AM (group 2) was not shown to increase the risk of recurrence (Table 4). In addition, the risk of recurrence with shorter duration of AM (group 2) remains statistically insignificant when combination AM and antenatal arrhythmia were added to the Cox multivariable analysis.

**Table 4:** Cox regression univariate and multivariate analysis for recurrence of SVT in infancy

	Crude HR (95% CI)	Adjusted HR (95% CI)	*p* value
Group 2 (2013–2017)	0.71 (0.34–1.45)	0.84 (0.40–1.77)	0.639
Sex (male)	0.91 (0.50–1.67)	0.96 (0.52–1.78)	0.902
WPW	2.21 (1.16–4.19)	2.55 (1.31–4.94)	0.006
Age at admission			< 0.001
< 1 week	1	1	
1–4 weeks	0.23 (0.08–0.65)	0.20 (0.07–0.59)	0.003
> 1 month	1.93 (1.00–3.70)	1.79 (0.91–3.50)	0.090

SVT, supraventricular tachycardia; HR, hazard ratio; WPW, Wolff-Parkinson-White syndrome

We performed a post hoc analysis in which the patients were divided into three groups based on AM duration: less than 6 months, 6 to 12 months, or more than 12 months (Supplementary Fig. 1). The 50-month recurrence-free survival rate remained unchanged between the groups.

**Fig. 2:**
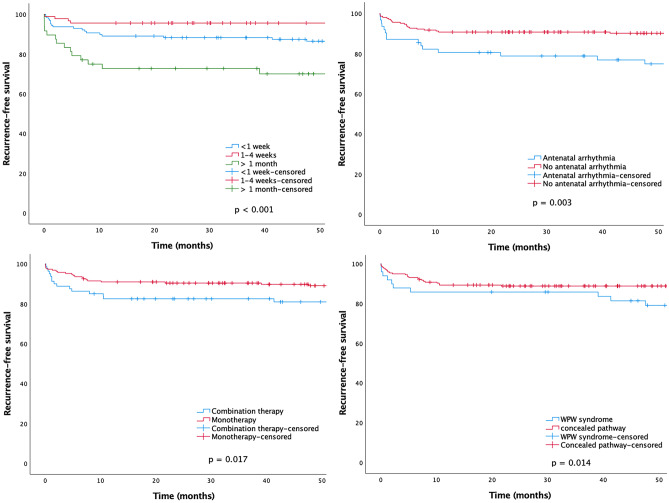
Recurrence-free survival after discontinuation of antiarrhythmic medication in infants with reentrant supraventricular tachycardia (*n* = 278) separated for age groups, antenatal arrhythmia, combination antiarrhythmic medication, and Wolff-Parkinson-White syndrome (WPW)

The recurrence of SVT after AM was associated with age older than 1 month at admission, antenatal arrhythmia, need of combination AM, and WPW syndrome (Fig. 2). Statistically independent risk factors for recurrence of SVT after discontinuation of AM were combination AM (*HR* 2.2, *95% CI* 1.14–4.20), WPW syndrome (*HR* 2.4, *95% CI* 1.25–4.59), and age over 1 month at admission (*HR* 2.2, *95% CI* 1.12–4.48, Table 5).

**Table 5:** Cox regression multivariate analysis for recurrence of SVT in infancy diagnosed between 2005 and 2017

	Crude HR (95% CI)	Adjusted HR (95% CI)	*p* value
Sex (male)	0.91 (0.50–1.67)	1.24 (0.65–2.37)	0.510
Antenatal arrhythmia	2.48 (1.34–4.60)	1.81 (0.93–3.56)	0.083
Combination AM	2.09 (1.14–3.85)	2.19 (1.14–4.20)	0.019
WPW syndrome	2.21 (1.16–4.19)	2.40 (1.25–4.59)	0.008
Age at admission			< 0.001
< 1 week	1	1	
1–4 weeks	0.23 (0.08–0.65)	0.26 (0.09–0.77)	0.015
> 1 month	1.93 (1.00–3.70)	2.24 (1.12–4.48)	0.023

SVT, supraventricular tachycardia; HR, hazard ratio; WPW, Wolff-Parkinson-White syndrome

## Discussion

This multicenter retrospective cohort study suggests that shortening AM prophylaxis duration in infants with AVRT did not increase the risk of subsequent SVT recurrence. Propranolol was sufficient as monotherapy for over half of the infants included in this study. Recurrences after discontinuation of AM were rare: 11% in those treated for 12 months and 10% in those treated for 6 months and in both groups during the 2-year follow-up period. The need of combination AM, WPW syndrome, and age over 1 month at diagnosis are associated with recurrence of SVT after discontinued AM. Infants who were diagnosed with SVT at age less than 1 month, who did not have WPW syndrome, and who had arrhythmia control with one AM were the least likely to have SVT recurrence after AM discontinuation.

We defined recurrence of SVT as an episode of SVT after discontinuation of AM. Breakthrough arrhythmias during AM were not documented as recurrences; instead, they were considered as inadequate therapy. Previous studies have reported a wide range of SVT recurrence rates, from 17 to 86% [3–5, 10–12] and shown that SVT resolves over time, and that relatively few patients present with recurrent SVT after the age of 12 months [3, 5, 12]. We found that 16% of infants with SVT had recurrence after AM was discontinued. Early breakthrough arrhythmia, which resulted in increasing the dose of the primary medication or adding another drug, was observed in one-third of the infants in this study. Furthermore, infants with AM combination therapy had a twofold higher risk of recurrence after discontinuation of AM compared to those who received a single AM. A single-center retrospective study on AM duration in infants with SVT had similar findings; infants with early breakthrough arrhythmia had higher risk of recurrence of SVT after discontinuation of AM [8]. The SAMIS study included infants aged less than 4 months without preexcitation and found no new recurrence of SVT during the follow-up between 6 and 12 months [6]. In fact, two other studies found that most breakthrough arrhythmias occurred within 3 months of AM initiation [6, 13]. In the present study, those infants with early breakthrough arrhythmias needing combination AM were most likely to have recurrence of SVT.

Previously, WPW syndrome has been reported in 9–39% of infants with reentrant SVT [5, 8, 12]. We found ventricular preexcitation in 19% of the patients and it was related to SVT recurrence, which aligns with the findings of previous studies [4, 11, 12]. Infants with ventricular preexcitation, even intermittently, had a 2.5-fold risk of recurrence after AM was discontinued. The ventricular preexcitation was more common in group 1 and annual incidence of ventricular preexcitation between 2005 and 2017 varied between 5 and 48%. However, there was no trend by the time or between the centers. Probably, the difference in incidence of WPW syndrome between the groups was related to a small cohort size. As preexcitation was more common in group 1 (treated with AM for 12 months) than in group 2 (treated with AM for 6 months), we included WPW syndrome in the multivariate model. In these analyses, it did not influence outcomes, even though it remained a significant risk factor for recurrence of SVT.

In this descriptive 12-year multicenter study, the treatment strategies were uniform between the centers. Propranolol was the first-line medication administered to over 90% of infants with SVT. An earlier study assessed the safety and efficacy of propranolol in 287 infants with SVT, and 254 had reentrant etiology [13]. Our findings are similar to those reported in that study: a high dose of propranolol controlled 67% of reentrant SVT episodes in hospital, and after discharge, 58% of infants continued to have their arrhythmia controlled [13].

The main difference between the two groups used in this study was the AM duration, which was the result of the study design. However, in real life, multiple factors influence management strategies. Hence, in group 2, the duration was not always restricted to 6 months. In addition, because historical controls were used, the overall follow-up time was longer in group 1 than in group 2. To address this AM duration issue, a post hoc analysis was performed in which the patients were divided into three groups based on AM duration: less than 6 months, 6 to 12 months, or more than 12 months. The 50-month recurrence-free survival rate remained unchanged between the groups.

The strength of our study is the unified nationwide protocol for the management of infants with SVT. Although our study comprised data from the five university hospitals in Finland, the management of patients with SVT did not substantially differ. However, the retrospective approach has several limitations. We were not able to access the medical records of 43 infants mostly because patient lived in different hospital catchment area and only acute treatment was given in the university hospital. The patient characteristics of infants who were lost to follow-up were not recorded and could not be compared with the patient cohort. In addition, even though we were able to collect up to 23 months of follow-up data for both groups, we were not able to follow all infants for a longer period. The study design included the use of historical controls, which is a weakness. The subtype of SVT was diagnosed based on ECG and as the differentiation between AVRT and AVNRT based on ECG remains uncertain, the very small proportion of infants with AVNRT may have been classified as having AVRT without preexcitation. The definition of breakthrough episodes during AM was lacking, and we were not able to evaluate the impact of early breakthrough episodes on recurrence of SVT after discontinuing AM. The patients with WPW syndrome mostly in group 1 were treated with longer AM, and, consequently, we are not able to confirm that this subgroup would benefit from shorter duration of AM. The cohort sizes were not based on power calculations; however, the recurrence-free survival time results were surprisingly similar between the two groups. It is reasonable to conclude that larger cohort sizes may not have changed this finding, which was the primary outcome of this study. Due to the retrospective design of the study, we were not able to systematically report the adverse effects of the medication. However, serious adverse effects were registered, and only one serious adverse effect related to acute amiodarone treatment was detected.

In conclusion, our study suggests that the duration of prophylactic AM can be safely shortened from 12 to 6 months in infants with SVT, without the risk of a higher SVT recurrence rate. Infants diagnosed at an older age, needing combination of AM, and having ventricular preexcitation have higher risk for recurrence after discontinuing AM. The further prospective controlled studies on duration of AM are needed.

## Supplementary Information

Below is the link to the electronic supplementary material.

Supplementary file1 (DOCX 27.7 KB)

Supplementary file2 (TIF 447 KB)

## Abbreviations

SVT: Supraventricular tachycardia
AM: Antiarrhythmic medication
IQR: Interquartile range
AVRT: Atrioventricular reentrant tachycardia
AVNRT: Atrioventricular nodal reentrant tachycardia
CHD: Congenital heart disease
ECG: Electrocardiogram
HR: Hazard ratio
WPW: Wolff-Parkinson-White
