# Supplementary material for: Multicenter cohort study on duration of antiarrhythmic medication for supraventricular tachycardia in infants
Source: Eur J Pediatr. 2022 Dec 28;182(3):1089–97. doi: 10.1007/s00431-022-04757-5 (PMC10023606; doi:10.1007/s00431-022-04757-5)
Supplement: Supplementary file 1 — Supplementary file1 (DOCX 27.7 KB) [file 431_2022_4757_MOESM1_ESM.docx]

Supplementary table 1. Types of congenital heart defects in infants diagnosed with SVT between 2005 and 2017 (n = 44).^1^

| Type of CHD | Number |
| --- | --- |
| VSD | 9 |
| TGA | 8 |
| IAA/HAA | 7 |
| Other^2^ | 3 |
| Ebstein anomaly | 3 |
| UVH | 3 |
| Truncus arteriosus | 2 |
| AS | 2 |
| TAPVD | 2 |
| ASD, secundum | 2 |
| AVSD | 1 |
| CoA | 1 |
| PDA | 1 |

SVT; supraventricular tachycardia, CHD, congenital heart disease; VSD, ventricular septal defect; TGA, transposition of the great arteries; IAA, interrupted aortic arch; HAA, hypoplastic aortic arch; UVH, univentricular heart; AS, aortic stenosis; TAPVD, Total anomalous pulmonary venous drainage; ASD, atrial septal defect; AVSD, atrioventricular septal defect; CoA, coarctation of the aorta; PDA, patent ductus arteriosus. ^1^ sorted hierarchically. ^2^ atrioventricular and ventriculoarterial discordance (n=1), bicuspid aortic valve (n=1), one case missing,
