# Supplementary figures and images for: Multicenter cohort study on duration of antiarrhythmic medication for supraventricular tachycardia in infants
Source: Eur J Pediatr. 2022 Dec 28;182(3):1089–97. doi: 10.1007/s00431-022-04757-5 (PMC10023606; doi:10.1007/s00431-022-04757-5)

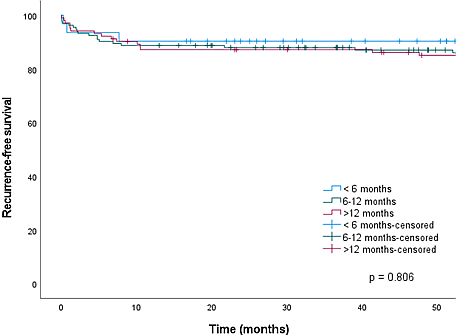

Supplement: Supplementary file 2 — Supplementary file2 (TIF 447 KB) [file 431_2022_4757_MOESM2_ESM.tif]
